# The protective effect and underlying mechanism of metformin on neointima formation in fructose-induced insulin resistant rats

**DOI:** 10.1186/1475-2840-12-58

**Published:** 2013-04-05

**Authors:** Jianxin Lu, Jingzhang Ji, Howard Meng, David Wang, Bo Jiang, Lixin Liu, Edward Randell, Khosrow Adeli, Qing H Meng

**Affiliations:** 1 Key Laboratory of Laboratory Medicine, Ministry of Education, Zhejiang Provincial Key Laboratory of Medical Genetics, School of Laboratory Medicine, Wenzhou Medical College, Wenzhou, China; 2 Department of Pathology and Laboratory Medicine, University of Saskatchewan, Saskatoon, SK, Canada; 3 Department of Laboratory Medicine and Pathobiology (D.W., H. M., K. A.), University of Toronto, Toronto, Canada; 4 Department of Pharmacology, University of Saskatchewan, Saskatoon, SK, Canada; 5 Laboratory Medicine, Faculty of Medicine, Memorial University, St. John’s, NL, Canada

**Keywords:** Neointimal hyperplasia, Insulin resistance, Methylglyoxal, Metformin, Rat

## Abstract

**Background:**

Insulin resistance is strongly associated with the development of type 2 diabetes and cardiovascular disease. However, the underlying mechanisms linking insulin resistance and the development of atherosclerosis have not been fully elucidated. Moreover, the protective effect of antihyperglycemic agent, metformin, is not fully understood. This study investigated the protective effects and underlying mechanisms of metformin in balloon-injury induced stenosis in insulin resistant rats.

**Methods:**

After 4 weeks high fructose diet, rats received balloon catheter injury on carotid arteries and were sacrificed at 1 and 4 weeks post injury. Biochemical, histological, and molecular changes were investigated.

**Results:**

Plasma levels of glucose, insulin, total cholesterol, triglyceride, free fatty acids, and methylglyoxal were highly increased in fructose-induced insulin resistant rats and treatment with metformin significantly improved this metabolic profile. The neointimal formation of the carotid arteries was enhanced, and treatment with metformin markedly attenuated neointimal hyperplasia. A significant reduction in BrdU-positive cells in the neointima was observed in the metformin-treated group (P < 0.01). Insulin signaling pathways were inhibited in insulin resistant rats while treatment with metformin enhanced the expression of insulin signaling pathways. Increased expression of JNK and NFKB was suppressed following metformin treatment. Vasoreactivity was impaired while treatment with metformin attenuated phenylephrine-induced vasoconstriction and enhanced methacholine-induced vasorelaxation of the balloon injured carotid arteries in insulin resistant rats.

**Conclusion:**

The balloon-injury induced neointimal formation of the carotid arteries is enhanced by insulin resistance. Treatment with metformin significantly attenuates neointimal hyperplasia through inhibition of smooth muscle cell proliferation, migration, and inflammation as well as by improvement of the insulin signaling pathway.

## Background

Insulin resistance is the key pathophysiological feature of obesity and type 2 diabetes. Emerging evidence suggests that insulin resistance is strongly associated with the development of atherosclerosis and cardiovascular disease [1,2]. There is a strong association between reduced insulin sensitivity and carotid intima-media thickness [1]. Moreover, insulin resistance enhances neointimal hyperplasia after balloon injury in rats [3-5].

Methylglyoxal (MG) is an intermediate endogenous product of glucose and fructose [6]. MG is highly elevated in diabetes and is associated with the development of diabetic complications [7-9]. It has been shown that MG administration induces atherosclerotic changes in rats [10]. Substantial evidence suggests that increased dietary consumption of fructose induces insulin resistance in animals and humans [11-13]. Fructose-induced insulin resistance has been well established and characterized in rats [12,14]. We and others have demonstrated that fructose is a major precursor of MG [6,15]. Therefore, there is likely an association between MG production and the development of fructose-induced insulin resistance and insulin resistance-associated atherosclerosis exists.

Metformin is an insulin-sensitizing agent with potent antihyperglycemic properties. These properties of metformin are mainly attributed to suppression of hepatic gluconeogenesis and to increased peripheral tissue insulin sensitivity [16]. Metformin serves as the scavenger of MG and reduces the rate of progression to type 2 diabetes in humans with obesity or impaired glucose tolerance [17]. Metformin has long been known to reduce the development of atherosclerotic lesions in animal models, and clinical studies have shown the drug to reduce surrogate measures such as carotid intima-media thickness [18].

However, little is known of the protective effects and mechanismas of metformin on the pathogenesis of atherosclerosis in insulin resistance. The aim of this study was to investigate the protective effects of metformin and the underlying mechanisms on balloon-injury induced stenosis in fructose-induced insulin resistance.

## Methods

### Chemicals

Unless otherwise specified, all chemicals were purchased from Sigma-Aldrich (St. Louis, Mo., USA).

### Experimental design

All experiments were performed following the guidelines of the Canadian Council on Animal Care and all experimental protocols were approved by the Animal Research Ethics Board at the University of Saskatchewan. After one week of adaptation with regular diet, male Sprague–Dawley (SD) rats (aged of 6 weeks and weighing 175 g) were randomly assigned to one of two different groups (n = 8 each): high fructose diet (60%, Harlan) (experimental) or normal chow diet (controls) with or without administration of metformin. Animals in the experimental group were fed a fructose-rich diet (60%; Harlan) for 4 weeks in order to induce insulin resistance [12,14]. In another group, metformin (300 mg/kg/day in 3 g/L in drinking water) was administered simultaneously throughout the experimental period to rats receiving the high fructose diet (60%; Harlan). Metformin was also given to a subgroup of rats receiving normal chow diet. Rats were housed in cages on a 12-hour light/dark cycle at ambient temperature (21-23°C), and relative humidity was held at 55 ± 10% in the colony room. Water was given *ad libitum*.

After 4 weeks of diet intervention, rats received carotid balloon injury and were sacrificed at 1 and 4 weeks post balloon injury. The above diet regime was continued until the end of the study.

### Carotid artery balloon injury

After the 4-week dietary intervention, the balloon denudation injuries were performed in the rat carotid artery following a previously described procedure [19]. Briefly, a 2F Fogarty balloon embolectomy catheter (Baxter Health Care Co, Toronto) was introduced into the left external carotid artery and further into the common carotid to denude the vessel of endothelium. Bromodeoxyuridine (BrdU) (100 mg/kg) was given intraperitoneally 18 hours before euthanization on day 7 after balloon injury for analysis of cellular proliferation in the various groups of rats. The animals were euthanized with an overdose of pentobarbital (200 mg/kg) at 1 or 4 weeks following balloon injury and the carotid arteries were collected for analyses.

### Metabolic measurements

After 10 hours of fasting, rats were anesthetised and blood pressure was measured. Fasting blood (1 ml) was collected through the abdominal vein to determine fasting glucose and insulin levels prior to administering 1 U insulin/kg of body weight via the portal vein for insulin tolerance testing. The animals were euthanized with an overdose of pentobarbital (200 mg/kg) 15 min after insulin was given and the blood was collected. Plasma glucose and lipid profiles were determined by enzymatic colorimetric techniques on Roche Cobas 6000 (Roche Diagnostics, USA). Plasma insulin was assayed using a commercially available immunoassay kit by ELISA (Mercodia, Upsala, Sweden). Non-esterified free fatty acids were measured using a method described previously in our laboratory [20]. Plasma MG was measured by liquid chromatography-tandem mass spectrometry (LC-MS/MS) [8].

### Histological and morphometric analyses

Histological and morphometric analyses were performed as described previously [19]. The carotid arteries were perfusion fixed at a constant physiologic pressure of 125 mmHg with 4% paraformaldehyde. The carotid arteries were carefully stripped of adventitia and excised between the origin at the aorta and the carotid bifurcation. The cross-sections of carotid artery were stained with hematoxylin and eosin and photographed. The intimal and medial cross-sectional areas of the carotid arteries were measured using an NIH Image 1.62 program and the intima/media ratios of the cross-sections were calculated.

### Immunohistological staining

After the collection of carotid arteries from the above, the carotid artery was fixed in 4% paraformaldehyde for 12 hours, embedded in paraffin and sectioned for immunohistostaining. Detection of smooth muscle cell (SMC) DNA synthesis was performed using a modified BrdU incorporation method as previously described [19]. Briefly, sections were incubated with monoclonal mouse antibody against BrdU (1/10 dilution, Cat. No. 1-299-964, Roche). Primary antibodies were detected using sheep anti-mouse-Ig-alkaline phosphatase (1/10 dilution). Hematoxylin was used for countstaining. Proliferation was determined by calculating the BrdU labeling index expressed as the ratio of BrdU-positive cells to total nucleated cells. Apoptosis was also assessed using a terminal deoxynucleotidyl transferase (TdT)-mediated dUTP nick end-labeling (TUNEL) apoptosis detection kit (Cat. No. 17–141, UPSTATE) [19].

### Smooth muscle cell culture and wound assay

Smooth muscle cells (SMCs) were isolated from the neointima of the injured carotid arteries of rats by enzymatic dispersion as described above [21]. To examine the role of fructose and MG on the SMC response to injury, we performed a scrape wound assay on confluent SMCs grown *in vitro* - an assay that mimics the cell migration and proliferation occurring during intimal hyperplasia [21]. Cells were grown to confluence in a 6-well tissue culture plate and then wounded with a P20 pipette tip to create a lengthwise wound measuring ~300 μm in width. Measurements of wound width were obtained every 24 hours over a period of 72 hours using Nikon NIS-elements BR3.1. Mean values were expressed as a percent of wound closure, which was calculated as follows: Percent wound closure = 1 − (width_t_/width_0_) × 100%.

### Matrix metalloproteinase activity

To investigate whether matrix metalloproteinase (MMP) could be involved, the effects of fructose and MG on MMP activities were assessed in an *in vitro* experiment. MMP activity was measured using gelatin zymography. In brief, medial and neointimal SMCs were harvested and seeded at a density of 60,000 cells/well in 96-well plates. The cells were incubated with 10% FCS for 8 hours to allow cell attachment. The cells were then serum starved for 16 hours, followed by incubation with various concentrations of fructose or MG in 10% FCS for 24 hours. Conditioned media from each well was used as a substrate for MMP activity as previously reported [22]. After electrophoresis, the gels were incubated and then stained with Coomassie blue (BioRad). MMP activity was evident as cleared bands of substrate lysis, with MMPs identified by their molecular weights. The intensity of the bands was quantitated by NIH image.

### Western blotting

The Western blotting was performed following a previously described procedure [20,23]. Since a large amount of protein is needed for Western blotting, the liver was used for determination of the expression of signalling molecules in insulin resistant rats. The rat livers were homogenized in a freshly prepared lysis buffer and centrifuged at 12,000g for 20 min at 4°C. Aliquots were run on 7.5-12% separating and 4% stacking gels and electro-transferred onto polyvinylidene fluoride membranes in transfer buffer. Dilutions (1:1,000) of primary antibodies to insulin receptor (IR), Tyr972-phosphorylated IR, insulin receptor substrate-1 (IRS-1), Tyr612-phosphorylated IRS-1, adenosine monophosphate-activated protein kinase (AMPKα), Thr172-phosphorylated AMPKα, and GAPDH (Cell Signaling Technology) were applied in blocking buffer. Goat anti-rabbit or goat anti-mouse secondary antibody (1:2,000 dilutions) (Santa Cruz Biotechnology) was applied and washed in TBS-Tween. Specific protein bands were detected using Western Lightning PLUS-ECL Reagents Plus (PerkinElmer Life and Analytical) and images were captured by an imaging system (ChemiDoc™ XRS + System, Bio-Rad). Relative band intensities were quantified using the Adobe Photoshop Program.

### Measurement of mRNA levels in SMCs *in vitro*

SMCs were isolated from the injured carotid arteries by enzymatic dispersion as described above [24]. SMCs were grown in DMEM supplemented with 10% FCS and 2% penicillin/streptomycin at 37°C with 5% CO_2_. Cells were treated with fructose or MG with and without metformin. Plating RNA was extracted by Trizol™ extraction (Invitrogen, Carlsbad, CA), treated with DNase I to remove contaminating genomic DNA (Fermentas), and reverse transcribed using the Superscript First-Strand Synthesis System for RT-PCR (Invitrogen). Quantitative real-time PCR (qRTPCR) reactions were performed using the ABI 7900 as described previously [24]. Primer sequences made by Invitrogen are listed below:

JNK-F: AAGCAGCAAGGCTACTCCTTCTCA, JNK-R: ATCGAGACTGCTGTCTGTGTCTGA, p65-NF-kB-F: CATGCGTTTCCGTTACAAGTGCGA, p65-NF-kB-R: TGGGTGCGTCTTAGTGGTATCTGT.

Each experiment was repeated independently 3 times, and mRNA samples were assayed in triplicate.

### Tissue contractility study

The tissue contractility study was conducted using a procedure described previously in our laboratory [19]. In brief, carotid arteries were removed and placed in cold physiological saline solution (PSS). The carotid rings were carefully dissected and the vessel segments (2.0 mm) were mounted in a four-channel wire myograph (Multi myogragh, Model 610M, Denmark) aerated with a gas mixture of 95%O_2_-5%CO_2_ at 37°C. Phenylephrine (PHE, 10^-8 ^to 10^-5 ^mol/L) was added cumulatively to generate a PHE dose–response curves. The endothelium-dependent vasodilator, methacholine (Mch 10^-9 ^to 10^-5 ^mol/L), was added cumulatively to generate the relaxation dose responses.

### Statistical analysis

Results are expressed as mean ± SD. All data were analyzed using analysis of variance (ANOVA). The difference between the variances of the two groups was compared using a two-tailed Student’s t test or one-way ANOVA followed by a post hoc analysis (Tukey test). Statistical significance was considered when P < 0.05.

## Results

### The effects of metformin on metabolic changes in insulin resistant rats

The metabolic changes determined at 4 weeks after balloon injury and following 4-week high fructose diet are summarized in Table 1. Increased body weight was observed in rats receiving high fructose diet, while treatment with metformin prevented the gain of body weight (Table 1). Blood glucose and insulin levels were elevated in rats on the high fructose diet and treatment with metformin improved glucose and insulin levels (Table 1). Plasma total cholesterol and triglyceride levels were significantly increased in the fructose group compared to the controls and treatment with metformin improved the lipid levels. LDL-cholesterol levels showed an increasing trend with time while on the fructose diet, but treatment with metformin reduced LDL-cholesterol levels significantly. Free fatty acid levels were also highly increased after fructose diet, but elevated free fatty acid levels were improved in rats following treatment with metformin (Table 1). Most notably, plasma MG levels from the high fructose group were significantly increased compared to those from the normal chow diet controls (513.33 ± 58.29 vs. 190.40 ± 52.94 nmol/L, P < 0.01). Treatment with metformin significantly reduced plasma MG levels (Table 1). There were no significant differences in metabolic changes between the chow group and the chow group received metformin (data not shown)**.**

**Table 1:** Effects of metformin on metabolic changes in fructose-induced insulin resistant rats

	**Chow control**	**Fructose diet (60%)**	**Fructose + Metformin**
Body weight (g)	514.0 ± 25.4	554.0 ± 30.5^*^	517.2 ± 15.0^†^
Liver weight (g)	13.8 ± 2.1	18.0 ± 1.0	14.4 ± 2.2
Heart weight (g)	1.5 ± 0.2	2.1 ± 0.3	1.6 ± 0.1
Kidneys weight (g)	3.1 ± 0.7	4.7 ± 0.9	3.3 ± 0.5
Mean BP (mmHg)	94.6 ± 10.9	93.1 ± 4.5	86.5 ± 9.2
Glucose (mmol/L)	5.80 ± 0.41	6.32 ± 0.53^*^	5.24 ± 0.69^‡^
Insulin (μg/L)	0.14 ± 0.02	0.55 ± 0.22^*^	0.28 ± 0.02^†^
Cholesterol (mmol/L)	3.26 ± 0.22	3.97 ± 0.18^*^	3.46 ± 0.22^†^
HDL-C (mmol/L)	1.33 ± 0.05	1.42 ± 0.23^*^	1.48 ± 0.29^†^
LDL-C (mmol/L)	0.97 ± 0.08	1.16 ± 0.12	0.82 ± 0.15^†^
Triglyceride (mmol/L)	2.10 ± 0.40	3.27 ± 1.02^**^	2.56 ± 0.75^†^
FFA (mmol/L)	0.77 ± 0.18	2.00 ± 0.31^**^	1.17 ± 0.22^‡^
MG (nmol/L)	190.40 ± 52.94	513.33 ± 58.29^**^	238.00 ± 42.00^†^

^*^P < 0.05 vs. Chow control, ^**^P <0.01 Fructose vs. Chow control.

^†^P < 0.05 vs. Fructose, ^‡^P < 0.01 vs. Fructose.

BP: blood pressure, HDL-C: high-density lipoprotein cholesterol, LDL-C: low-density lipoprotein cholesterol, FFA: free fatty acids, MG: methylglyoxal.

Determinations were performed 4 weeks after balloon injury following 4 week high fructose diet.

### Metformin treatment attenuates neointimal formation in fructose-induced insulin resistant rats

**Figure 1:**
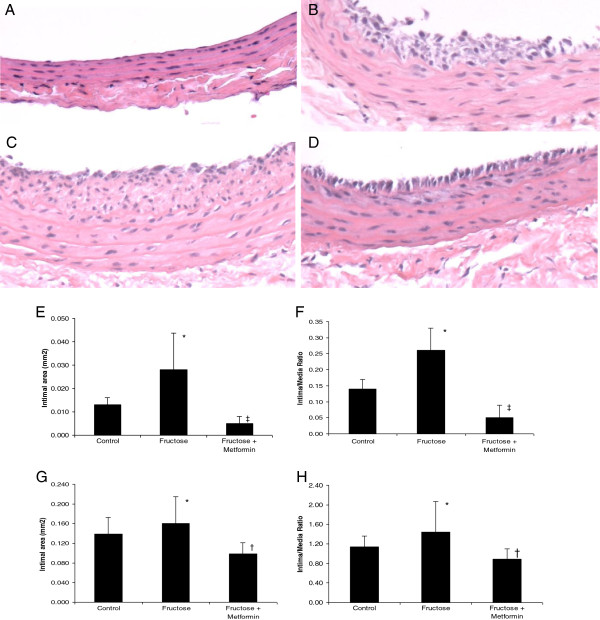
**Histological and morphometric changes of balloon injury-induced neointimal formation in insulin resistant rats. A**) A representative of the uninjured carotid artery in normal control rats. **B**) A representative of histological change 4 week after balloon injury, showing neointimal hyperplasia in chow group treated with metformin. **C**) A representative of histological change of carotid artery 4 weeks after balloon injury, showing severe neointimal hyperplasia in rats with high fructose diet. **D**) A representative of histologic change 4 weeks after balloon injury in rats with high fructose diet and metformin administration (H&E staining, magnification x 100). Morphometric analyses on intimal area and intima to media ratio were performed to quantitate neointimal hyperplasia at week 1 (**E** and **F**) and week 4 (**G** and **H**). ^*^P < 0.05 vs. Chow control, ^†^P < 0.05 vs. Fructose group.

**Figure 2:**
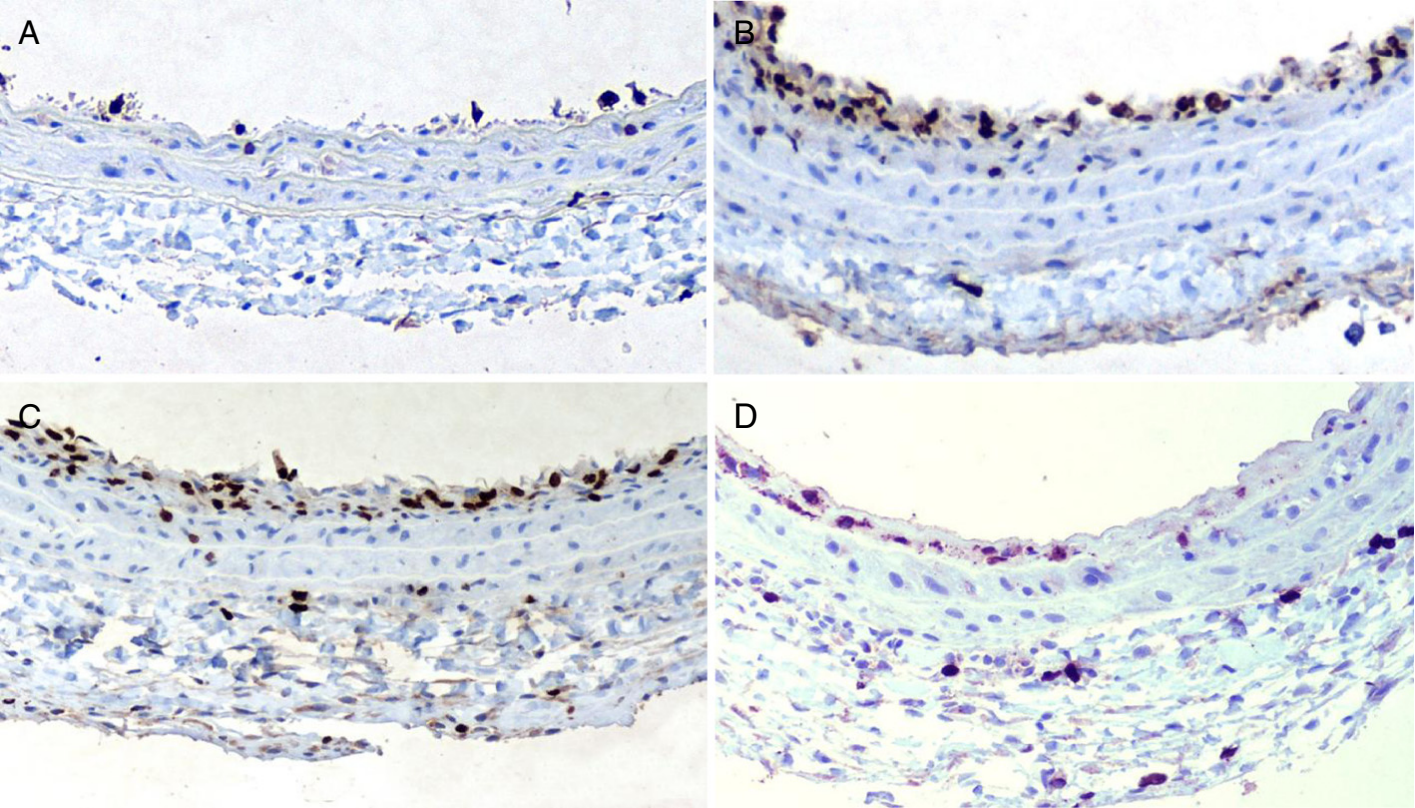
**Antiproliferative effect of metformin on vascular smooth muscle cells.** BrdU-labeled nuclei are stained as dark brown. **A**: Uninjured; **B**: Injured control; **C**: Fructose; **D**: Fructose + Metformin.

Neointimal hyperplasia occurred following balloon injury (Figures 1A, B, and C). Neointimal hyperplasia remained evident though metformin was administered to the group of rats receiving chow diet (Figure 2B). The neointimal hyperplasia of the carotid arteries (Figure 1D) was dramatically inhibited following treatment with metformin compared with the untreated high fructose-fed rats at 4 weeks after balloon injury (Figure 1C). Morphometric analysis indicated that treatment with metformin significantly inhibited neointimal formation (0.010 ± 0.003 mm^2^, cross-sectional area) in the carotid arteries compared with untreated injured carotid arteries (0.03 ± 0.02 mm^2^, P < 0.05, n = 8), determined 1 week after balloon injury (Figure 1E). The intima/media ratio was also significantly reduced at 1 week after balloon injury (0.26 ± 0.07 vs. 0.05 ± 0.04, P < 0.01) (Figure 1F). Similar inhibitory effects on neointimal formation remained at 4 weeks after balloon injury in the metformin-treated compared with untreated groups (Figure 1G). The intima/media ratio was also decreased at 4 weeks after balloon injury (Figure 1H). There were no significant changes in media area among the three groups at the different time points. A chow control group also received balloon injury and showed neointimal hyperplasia but to a lesser degree compared to the fructose treated group. Treatment with metformin did not show significant attenuation.

### Antiproliferative effects of metformin on vascular SMCs

SMC proliferation was determined 7 days after carotid artery injury (Figures 2A and B). Treatment with metformin induced a significant reduction in the percentage of BrdU-positive cells in the neointima compared with vehicle controls (18.4 ± 2.8% vs. 43.5 ± 5.2%, P < 0.01, n = 6) (Figure 2D vs. Figure 2C). There was no significant difference of BrdU-labeled positive cells in media area between the two groups (4.7 ± 0.6% vs. 5.8 ± 0.9%, P > 0.05). Using TUNEL staining, we could not detect any significant change in the number of apoptotic cells in the neointima between the two groups (data not shown).

### The effect of metformin on SMC migration

The *in vitro* scratch assay revealed that both fructose and MG significantly enhanced SMC migration compared to the control group, as determined by wound closure at 24 hours (Table 2). The effect of fructose and MG on SMC migration also behaved in a dose-dependent manner (data not shown). Treatment with metformin for 24 hours and 48 hours significantly attenuated the effects of fructose and MG on wound closure (P < 0.01). Data were triplicates with three independent experiments.

**Table 2:** Metformin inhibits fructose and MG-induced intimal SMC migration

**Compound**		**Wound closure (%)**	
	**0 h**	**24 h**	**48 h**
Control	0	28.5 ± 1.3	82.2 ± 5.30^**^
Fructose (30 mM)	0	54.1 ± 1.8	85.3 ± 1.9^**^
Fructose (30 mM) + Metformin (10 mM)	0	23.6 ± 1.1	73.6 ± 1.4^*^
MG (100 μM)	0	83.4 ± 0.9	96.7 ± 1.4^*^
MG (100 μM) + Metformin (10 mM)	0	22.2 ± 6.5	66.4 ± 6.9^*^

^*^P < 0.05 vs. 24 hr, ^**^P < 0.01 vs. 24 hr.

### The effect of metformin on MMP activity

**Figure 3:**
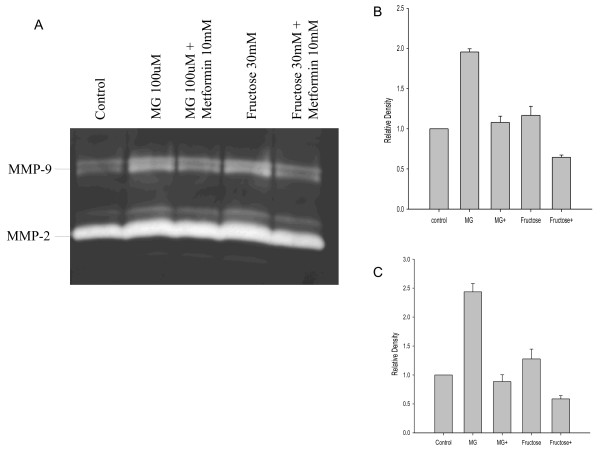
**Effects of metformin on MMP activity.** Enhanced MMP activities were seen in intimal SMCs treated with MG or fructose *in vitro* (Figure 3**A**). Treatment with metformin significantly inhibited the enzyme activity as observed through the relatively faint band and the reduced intensity of the bands quantified by NIH image. Figures 3**B** and **C** indicate the relative density of MMP2 and MMP, respectively. MG + or Fructose + in Figures 3**B** and **C** means co-treatment with metformin.

Gelatin zymography showed enhanced MMP activities as indicated by high activity of both MMP-9 (the top band, 100 kDa) and MMP-2 (the lower band, 70 kDa) in intimal SMCs treated with MG or fructose. Treatment with metformin inhibited the MMP enzyme activities as evidenced by the relatively faint bands seen in the treated versus the untreated condition and the intensity of the bands quantified by densitometer (P < 0.01 for MG + metformin vs. MG and Fructose + metformin vs. Fructose, respectively) (Figure 3A, B, and C). Similar MMP activities and inhibitory effects of metformin on MMP activities were also observed in medial SMCs treated with MG or fructose (data not shown).

### Expression of insulin signalling and inflammatory markers in fructose-induced insulin resistant rats

**Figure 4:**
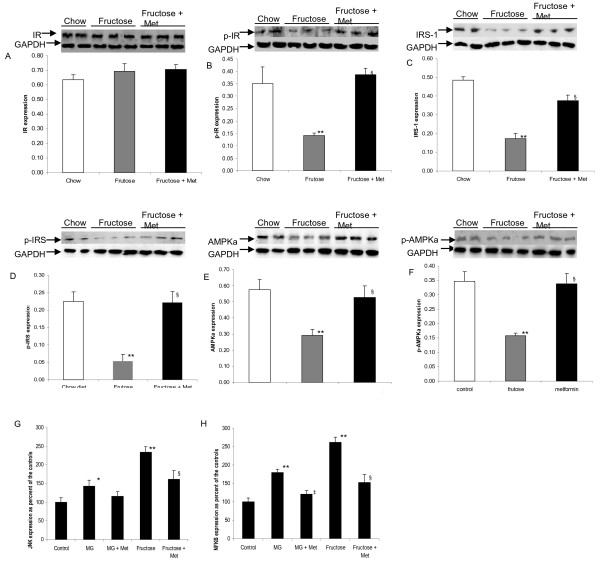
**Expression of insulin signalling and inflammatory markers in fructose-induced insulin-resistance in rats.** There was no change in IR expression (Figures 4**A**). Significant inhibition of p-IR, AMPK, p-AMPK, IRS-1, p-IRS was revealed in the liver in insulin resistant rats. Treatment with metformin reversed the inhibitory effects and improved the expression of these molecules (Figures 4**B**, **C**, **D**, **E**, and **F**). Real-time PCR shows increased expression of JNK and NFKB in MG or fructose treated SMCs (Figure 4**G** and **H**). Treatment with metformin attenuated this effect (Figures **G** and **H**). ^*^ P < 0.05, ^**^ P < 0.01 vs. the Chow control, ^‡^P < 0.01 vs. MG group, ^§^P < 0.01 vs. Fructose group.

There was no change in IR expression (Figure 4A) but the phosphorylation of IR was decreased in the liver (Figure 4B) following the 60% fructose diet and the development of insulin resistance. Significant inhibition of IRS-1, AMPKa, and their corresponding phosphorylated forms were observed in the liver by western blotting (Figures C, D, E, and F). Treatment with metformin reversed the inhibitory effects and significantly improved the expression of these molecules including phosphorylated IR (P < 0.01) (Figures 4A, B, C, D, E, and F). There were no significant differences in expression of these above signalling molecules between the chow group and the chow group received metformin (data not shown). In addition, using an *in vitro* assay, real-time PCR revealed that the expression of inflammatory markers JNK and NFKB was markedly increased in MG or fructose treated SMCs. Treatment with metformin attenuated this effect on JNK and NFKB in SMCs (Figures 4G and H).

### Improved vasoreactivity of carotids arteries by metformin in insulin resistant rats

**Figure 5:**
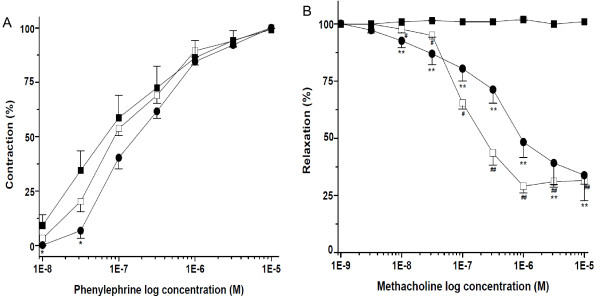
**Improved vasoreactivity of carotids arteries by metformin in fructose-induced insulin-resistant rats. A**) The vasocontraction of the carotid artery in response to phenylephrine increased 4 weeks after balloon injury compared with uninjured controls in insulin-resistant rats (■ injured carotids, □ uninjured carotids). Treatment with metformin attenuated the contraction (● ). **B**) Vasorelaxation was impaired and treatment with metformin improved methacholine-induced vasorelaxation (■ injured without metformin, □ uninjured carotids, ● injured with metformin) (n = 6 per group), ^*^P < 0.05 vs. fructose group with injury, ^**^P < 0.01 vs. fructose group with injury, ^#^P < 0.05 vs. fructose group with injury, ^##^P < 0.01 vs. fructose group with injury.

The vasocontraction of carotid artery, in response to phenylephrine, increased in the experimental group 4 weeks after balloon injury as compared with uninjured controls, though it did not achieve statistical significance (Figure 5A). Treatment with metformin attenuated PHE-induced vasoconstriction of the carotid artery compared with that of the untreated rats (EC_50_ = 244 ± 29 nmol/L vs. EC_50_ = 159 ± 27 nmol/L) (Figure 5A). The vasorelaxation of the carotid artery was severely impaired in fructose-fed rats 4 weeks after balloon injury compared to the uninjured controls (IC_50_ = 165 ± 7.6 nmol/L for the uninjured control) (Figure 5B). Interestingly, treatment with metformin improved methacholine-induced vasorelaxation in balloon injured carotid arteries compared with untreated balloon injured carotids (IC_50_ = 566 ± 44 nmol/L) (Figure 5B).

## Discussion

High fructose intake has been shown to induce insulin resistance, weight gain, and hyperlipidemia in animals and humans [11]. In our study, insulin resistance developed in SD rats following 8 weeks of feeding on a high fructose diet. The animals experienced characteristic changes that include increased body weight, glucose intolerance, increased serum insulin levels, and dyslipidemia. Treatment with metformin significantly improved these metabolic abnormalities. The observed effects of metformin are supported by the evidence that metformin decreases hepatic production of glucose and increases hepatic insulin sensitivity and glucose clearance [25,26].

Insulin resistance is strongly associated with the development of atherosclerosis and cardiovascular disease [1,2,27]. In our study, severe neointimal hyperplasia developed following balloon injury in insulin-resistant rats compared to normal chow controls. The neointimal hyperplasia observed was consistent with neointimal SMC proliferation, suggesting SMC proliferation contributed largely to the increased neointimal formation [28,29]. The neointimal hyperplasia of the carotid arteries was dramatically inhibited following treatment with metformin as compared with the untreated high fructose-fed rats those were determined at 1 week and 4 weeks after balloon injury. Neointimal SMC proliferation indicated by BrdU-positive cells was also inhibited in the group receiving metformin treatment. Metformin is known to reduce the development of atherosclerotic lesions and attenuate carotid intima-media thickness [18], but this is the first time that a similar effect on balloon injury-induced neointimal hyperplasia in insulin resistant rats has been demonstrated. Moreover, our *in vitro* scratch assay suggests that both fructose and MG-induced SMC migration may contribute to the neointimal formation and the inhibitory effect of metformin on SMC migration could contribute beneficial effects by reducing stenosis. Our *in vitro* experiment demonstrates that MMP activities were significantly increased in fructose or MG-treated SMCs. Treatment with metformin inhibited MMP activities. Previous studies have shown that MMP activity plays a major role in matrix accumulation and neointimal formation [24,30]. The observed inhibitory effect on MMP activity and the upregulation of AMPK expression by metformin in our study may suggest that the suppressive effect on MMP is AMPK-dependent [31]. Taken together, the increased SMCs proliferation, migration, and MMP activity induced by fructose are likely to contribute to the severe neointimal formation induced by balloon injury in insulin-resistant rats. Thus, metformin attenuated neointimal formation **is** mediated through inhibition of SMCs proliferation, migration, and MMP activity.

Plasma MG levels were highly increased in fructose-fed rats, while treatment with metformin dramatically decreased the MG levels. This effect can be attributed to metformin’s action as a scavenger of MG or through normalization of metabolic abnormalities [17]. In a previous study, we have shown that fructose is the precursor of MG [15]. Early research has also demonstrated that MG induces insulin resistance and that treatment with MG scavengers attenuates this effect in SD rats [32,33]. Studies have shown that MG induces an inhibitory effect on phosphorylation of IRS-1, PI3K activity, and insulin-stimulated phosphorylation of protein kinase B (PKB) and extracellular-regulated kinase ½ (ERK½) [23]. Metformin sensitizes insulin signalling through an AMP-activated protein kinase (AMPK)-mediated phosphatase and tensin homolog (PTEN) down-regulation [34]. Our findings show that the expression of AMPKa and IRS-1 was significantly down regulated in the liver in fructose-induced insulin-resistant rats while treatment with metformin reversed this inhibitory effect. These findings are consistent with metformin improving insulin signalling probably through lowering of MG levels. Increased expression of JNK and NFKB in SMCs suggests that inflammation may play a role in the pathogenesis of stenosis in fructose-induced insulin resistance and metformin plays a protective role through the inhibition of inflammatory pathway in insulin resistance and stenosis.

Vasorelaxation was severely impaired in balloon-catheter-injured carotid arteries in insulin-resistant rats. Nevertheless, vasorelaxation was significantly restored in rats that received treatment with metformin. We and others have demonstrated that impairment of endothelium-dependent vasorelaxation may be mediated by MG while metformin treatment significantly improves endothelial function and vasorelaxation [8,18,35-37]. These findings suggest that the improvement of endothelial function and vessel reactivity may be attributed to the effects of metformin on reduction of MG levels and improvement of insulin sensitivity [38]. Another study demonstrated that metformin reduces catecholamine-induced vasoconstriction through endothelium dependent and independent mechanisms in rats [39]. Demonstration of the vasoprotective effect of metformin in an insulin resistant model has not been previously reported.

In conclusions, this study has demonstrated that balloon-injury-induced neointimal formation of the carotid arteries is facilitated by fructose-induced insulin resistance. Treatment with metformin significantly attenuates balloon-injury induced neointimal hyperplasia through inhibition of SMC proliferation, migration, MMP activities, and inflammation, as well as by improvement of the insulin signaling pathway.

## Competing interests

The authors declare that they have no competing interests.

## Authors’ contributions

JL participated in study design, biochemical experiments, data interpretation, and manuscript preparation. JJ performed animal study and molecular analysis. HM conducted biochemical and molecular analyses. DW performed histological and molecular analyses. BJ performed animal surgery and vasoreactivity study. LL involved in experiment design, biochemical analysis, and data interpretation. ER performed biochemical analysis and data interpretation. KA participated in experiment design, biochemical analysis, and manuscript preparation. QHM involved in experiment design, biochemical analysis, data analysis, and manuscript preparation. All authors read and approved the final manuscript.
